# Experience of Alternative Technique for Branch Cannulation in Branched Endovascular Aneurysm Repair

**DOI:** 10.3390/jcm15072497

**Published:** 2026-03-24

**Authors:** Daniela Mazzaccaro, Paolo C. Righini, Fabiana Fancoli, Alfredo Modafferi, Giovanni Malacrida, Marina Galligani, Giovanni Nano

**Affiliations:** 1 Operative Unit of Vascular Surgery, IRCCS Policlinico San Donato, San Donato Milanese, 20097 Milan, Italy; 2 Department of Biomedical Sciences for Health, University of Milan, 20122 Milan, Italy

**Keywords:** thoraco-abdominal, aneurysm, B-EVAR, branch cannulation

## Abstract

**Background/Objectives**: To describe our experience of using an alternative technique for retrograde branch graft cannulation during Branched EndoVascular Aortic Repair (B-EVAR) of complex abdominal and thoraco-abdominal aortic aneurysm (TAAA) with branched endografts. **Methods**: Data from patients who underwent B-EVAR for TAAA/complex abdominal aneurysms and in whom the cannulation of a branch was performed through a retrograde approach with our technique were retrospectively collected and analyzed. The technique consists of the placement of two 4F Ber catheters in both femoral arteries, which are advanced in parallel with the main graft before its deployment and left in the aneurysmal sac. These catheters are then used as a bailout for the retrograde cannulation of one or more branches of the endograft. **Results**: The technique was employed in 4 patients (1 female, 1 urgent) treated from 2018 onward, allowing the successful catheterization of 4 challenging branches that could not be catheterized using other approaches. The patients’ mean age was 80.7 + 2.2 years. No intraoperative nor postoperative complications linked to the technique occurred. **Conclusions**: The placement of two catheters from both femoral arteries inside the aneurysmal sac before graft deployment can be safe and useful for bailout retrograde cannulation of any branch of the endograft, when other techniques fail.

## 1. Introduction

The use of fenestrated and branched endografts has progressively spread over the last decade for the treatment of pararenal and thoraco-abdominal aortic aneurysms, enabling the treatment of increasingly complex pathologies [1] with low mortality and morbidity rates compared to open surgery [2]. Moreover, with the advent of “off-the-shelf” endografts, which have been developed based on the anatomical variability of renovisceral vessels [3], urgent cases can also be treated.

Despite their lower invasiveness, however, the use of these types of endografts requires advanced endovascular skills due to the complex anatomy of the lesions [4].

In most cases, technical difficulties may arise during the completion of bridging stent-grafting of the involved renovisceral target vessels.

Given the presence of downward-oriented branches in the majority of available “off-the-shelf” and custom-made branched endografts, selective catheterization of these branches and the target vessels is usually performed through an antegrade approach via the trans-axillary way. However, prolonged manipulation of the aortic arch through the axillary access using a huge (>8F) sheath for challenging branch and vessels cannulation may increase the risk of perioperative complications [5]. When difficulties arise, some bailout techniques may be used [6,7,8,9,10,11].

The aim of our paper was to describe our experience of using an alternative technique for retrograde branch graft cannulation during Branched EndoVascular Aortic Repair (B-EVAR) of complex abdominal and thoraco-abdominal aortic aneurysms (TAAA) with off-the-shelf and custom-made branched endografts.

## 2. Materials and Methods

The study was conducted in accordance with the Declaration of Helsinki. Patients’ consents were all obtained for the anonymous publication of these data.

Data of patient who underwent B-EVAR of TAAA/complex abdominal aneurysms with off-the-shelf and custom-made branched endografts and in whom the cannulation of a branch was performed through a retrograde approach with our alternative technique were retrospectively collected and analyzed from an internal database (study approved by the Ethics Committee of San Raffaele Hospital, protocol code 113/INT/2014, approval date 11 December 2014).

In particular, age, sex, and patients’ comorbidities were recorded. The type of pathology and the diameter of the aneurysm were also recorded.

Intraoperative data included the type of endograft used (either “off-the-shelf” or custom-made), the target branch cannulated with our technique, total procedural time and fluoroscopy time, and the volume of administered contrast medium. Intraoperative complications and in-hospital complications were recorded as well, along with the occurrence of vessels’ access complications.

A statistical descriptive analysis was performed using Stata^®^16.1 (Stata Corp LLC, College Station, TX, USA).

Data were expressed as mean ± SD with 95% confidence intervals, or as proportions.

### The Technique

The bailout technique of side branch cannulation is similar to that already described by D’Elia et al. [6].

After a small surgical groin incision, the right common femoral artery is exposed for the main access. Then, a double arterial puncture is performed to place a 22F sheath for the branched main graft and a parallel 4F sheath. A percutaneous femoral access is performed on the left side, placing a 10F sheath after pre-positioning a Perclose ProGlideTM Suture (Abbott Vascular Inc., Santa Clara, CA, USA). Also, a small subclavicular left cutdown is performed to expose the axillary artery for the antegrade placement of a 90 cm long 8F sheath.

**Figure 1:**
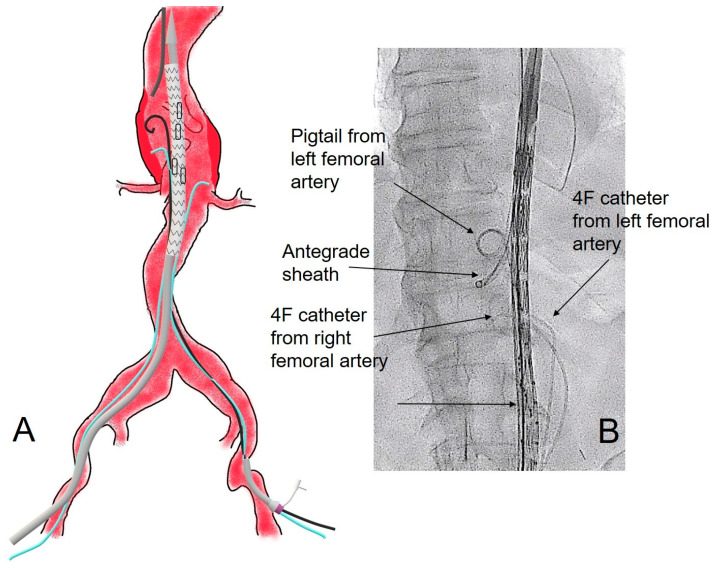
(**A**), on the left side, with a picture showing the placement of the branched endograft with the delivery system, still not deployed, and a parallel 4F Ber catheter left in the aneurysmal sac in front of the right renal artery, from the right femoral access. On the left femoral access, a 4F Pigtail catheter for aortography and a 4F Ber catheter were placed in parallel in the aneurysmal sac in front of the left renal artery, both through the 10F sheath. From the axillary access above, a long 8F gray sheath is placed in the visceral aorta. (**B**), on the right side, showing the respective fluoroscopic image during the procedure.

From the right femoral access, the branched main graft is advanced through a superstiff precurved guidewire (Lunderquist, Cook Medical, Bloomington, IN, USA) in the 22F sheath, and a 4F Ber catheter (Cordis, Bridgewater, NJ, USA) is advanced through the 4F sheath and left in parallel in the aneurysmal sac in front of the right renal artery. On the left side, a 4F Pigtail catheter (Cordis, Bridgewater, NJ, USA) is placed in the thoracic aorta for the aortography, and a 4F Ber catheter (Cordis, Bridgewater, NJ, USA) is placed in parallel in the aneurysmal sac in front of the left renal artery (Figure 1), both through the 10F sheath.

**Figure 2:**
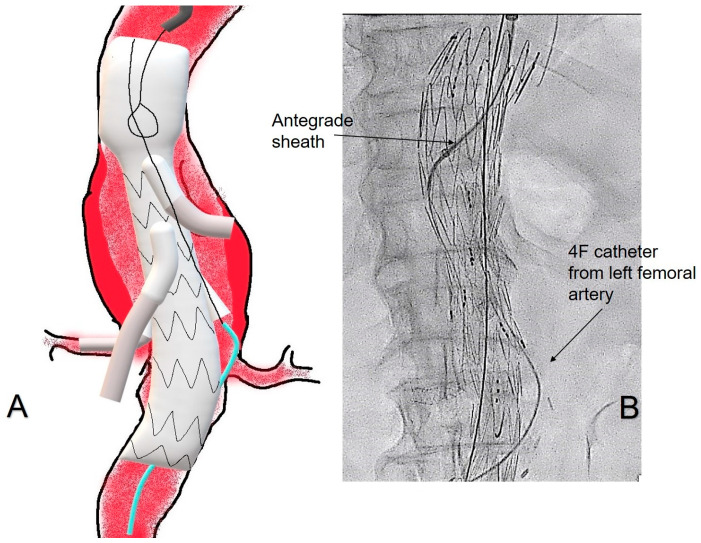
(**A**), on the left side, with a picture showing a branched endograft, which has been deployed, and bridging stenting of the celiac trunk, the superior mesenteric artery and the right renal artery. The azure 4F Ber catheter is in front of the left renal branch. After the successful retrograde catheterization of the latter, the guidewire inside the Ber is captured from the antegrade axillary sheath using a Gooseneck snare. (**B**), on the right side, showing the respective fluoroscopic image during the procedure.

After placement of the branched main graft, the 4F Pigtail catheter is retrieved from the aneurysmal sac and placed inside the endograft for diagnostic angiography. Then, the branches are selectively cannulated through the antegrade axillary approach, as well as their respective target vessels, using a 0.035” Rosen (Cook Medical, Bloomington, IN, USA) guidewire inside a catheter with an optimal curvature, and bridging stenting is performed with GORE^®^ Viabahn^®^ VBX (Gore, Flagstaff, AZ, USA) stent-grafts. When the antegrade axillary approach is challenging and unsuccessful after multiple attempts and steerable catheters are not available (i.e., in urgent cases) or fail, the bailout technique is used. Therefore, from one of the 4F Ber catheter placed inside the aneurysm sac, a 0.014” guidewire is advanced inside the side branch, and then the guidewire is captured from the antegrade axillary sheath using a Gooseneck snare (Figure 2).

**Figure 3:**
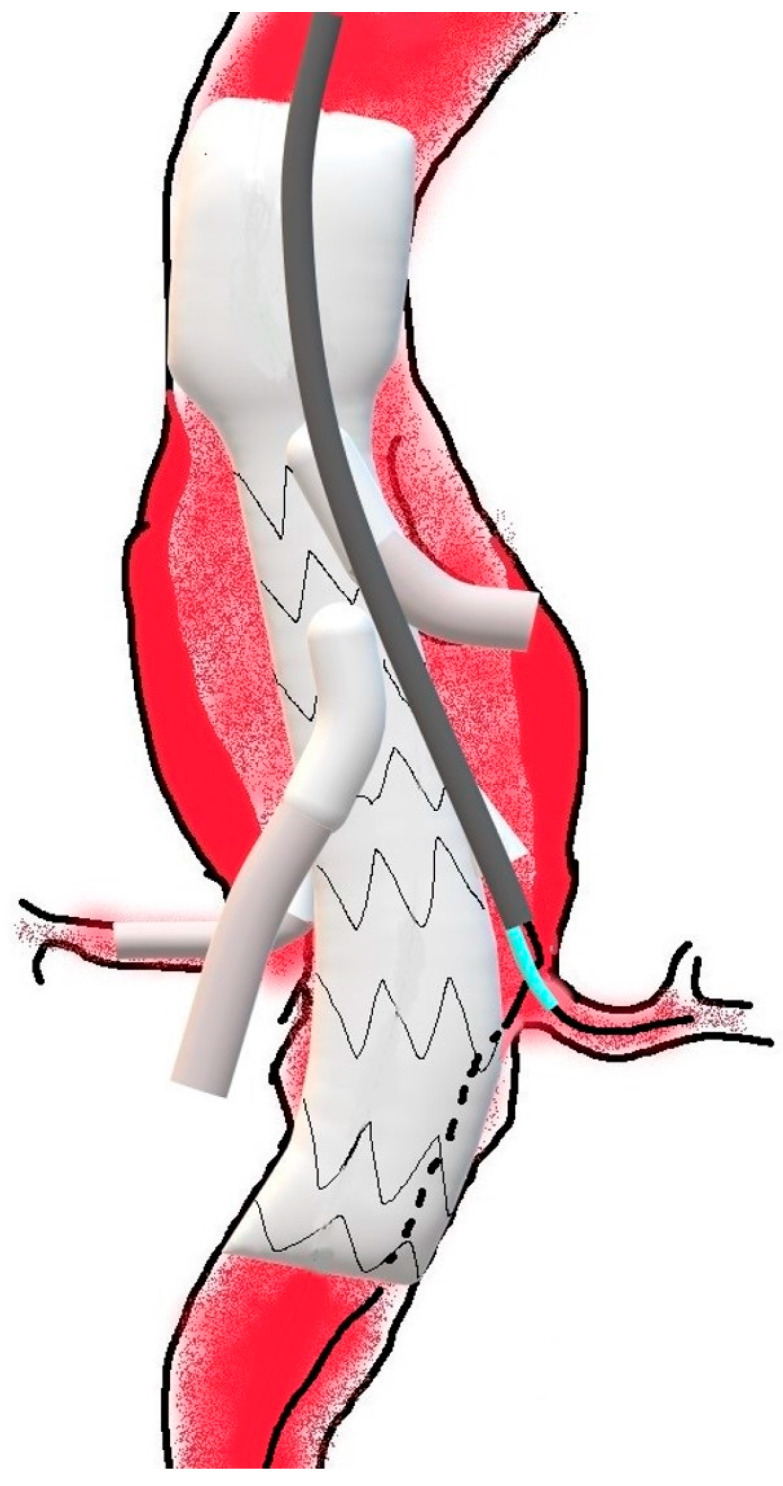
Picture showing the 8F long gray sheath that was successfully advanced from the axillary artery through the left renal branch in the aneurysmal sac, in front of the left renal artery on the “through-and-through” axillary-femoral guidewire, which is still in the aneurysmal sac outside the graft (dotted part). In parallel, the azure 4F Ber catheter is advanced over the hydrophilic guidewire, which can be used to cannulate the left renal artery.

On this “Through-and-Through” axillary-femoral guidewire, the 8F long axillary sheath is advanced from the axillary artery through the side branch in the aneurysmal sac. Then, from the axillary access, a 0.035” hydrophilic guidewire is advanced in parallel, the corresponding target vessel is cannulated (Figure 3), and the procedure is completed with bridging stenting of the remaining target vessel. Then, both 4F Ber catheters from the femoral accesses are removed.

## 3. Results

From 2018 onward, 4 patients out of 6 B-EVAR (1 female, 25%; mean age 80.7 ± 2.2 years) were successfully treated with either “off-the-shelf” (2 patients, 50%) or custom-made branched endografts for pararenal (2 patients, 50%) and thoraco-abdominal aortic aneurysms (Table 1) using our technique for retrograde outer-to-inner catheterization of a challenging branch. In the remaining 2 patients, the technique was avoided due to small-caliper access vessels.

In most cases, the difficulties arose for the catheterization of the renal branches (left renal branch 2/4, 50%; right renal branch 1/4, 25%) due to their malrotation after graft deployment.

Total procedural time was 222.5 ± 21.9 min, while fluoroscopy time was 106.2 ± 5 min. The mean total amount of diluted contrast medium used (1:3 with saline solution) was 215.7 ± 20.8 mL (Table 2).

No intraoperative complications occurred (Table 2), nor access-related complications. During the in-hospital stay, the patient treated for the urgent (symptomatic) pararenal AAA developed a myocardial infarction that required percutaneous descending coronary artery revascularization on postoperative day 3, but then he was discharged in good clinical condition after 7 days. A transient paraplegia also occurred but was resolved with cerebrospinal fluid drainage. Renal failure requiring permanent dialysis occurred in a patient who was treated for a Crawford type II TAAA and had preoperative renal failure. The branches to the renal vessels were, however, patent.

Mean follow-up was 54.5 ± 23.7 months, during which neither endoleaks nor branch or target vessels’ occlusions occurred.

## 4. Discussion

“Off-the-shelf” and custom-made branched endografts are increasingly used for the treatment of complex abdominal and thoraco-abdominal aortic aneurysms. Satisfactory immediate results have been reported up to now. Nevertheless, intraoperative technical failure can occur, mainly due to the inability to complete the bridging stenting of the target vessels [8,12].

When using “off-the-shelf” endografts, this issue can be the consequence of the fact that the branches of these devices are not built on the patient’s specific anatomy, but they fit half to a quarter of the population’s anatomies [13]. The endograft outer branch is downward-oriented, which makes it easy to cannulate them via an antegrade approach. Nevertheless, the target artery orifice, particularly of the renal arteries, may originate upward-oriented or perpendicular to the longitudinal axis of the aorta. In such cases, a retrograde femoral cannulation could be selected. Further technical difficulties may arise during the deployment of the endograft, caused, for example, by the twisting of the device with consequent imperfect positioning toward respective target vessels, or in very rare cases, by incomplete opening of the graft branches.

Therefore, when cannulation of the branches becomes challenging and time-consuming [14] or when difficulties arise for malrotation/misdisplacement of the graft or compression of the branches, some bailout techniques could be used.

Makaloski et al. [5], for example, reported the use of a manually steerable sheath from a transfemoral access as a safe and effective alternative to upper extremity access for retrograde cannulation of the branches and target vessels.

When manually steerable catheters fail or are not immediately available, as for urgent cases, our technique can be considered as a valuable and safe alternative, with no adjunctive complications due to a possible open approach, and a paltry cost added.

Indeed, our technique was similar to that described by D’Elia et al. [6] as a bailout maneuver for challenging cannulation of a branch of the left renal artery in the treatment of a type III TAAA. They used a 7F sheath over an Amplatz 0.035” guidewire that was advanced first in the diagnostic pigtail catheter in the aneurysm sac between the branched endograft and a previous tubular aortic graft, to cannulate the branch for the left renal artery from outside to inside. Then, the guidewire was snared from above and on this “through-and-through” guidewire, the axillary sheath was advanced in the left renal branch to complete the bridging stenting.

Our technique is different, first because the bailout catheter for retrograde outer-to-inner branch cannulation has a smaller caliper (4F), giving the advantage of better navigability with a lower profile. Second, we use two catheters for each side, while the pigtail catheter is independent and can still be used for diagnostic control, especially when rapidly needed during branch cannulation.

One may argue that a single Ber catheter could be enough as a bailout, but in our experience, the use of both catheters can be useful when there is not much space left between the endograft and the aneurysm sac, and moving the catheter from one side to the other in this space could be challenging or even not feasible. Indeed, D’Elia et al. [6] underlined that the diameter of the aneurysm sac at the level of their maneuver was very large (10 cm), while our technique can also be employed for smaller diameters.

Also, Antonello et al. [11], based on D’Elia’s bailout technique, reported their anecdotical experience of the “double-snare” technique in a very challenging case in which the retrograde outer-to-inner branch cannulation was performed from the left femoral access after snaring in the aneurysm sac a guidewire coming from the right side inside the graft. Then, after advancing the catheter from the left femoral access from outer to inside the branch and then in the aortic segment, a second snare coming from the axillary access captured the guidewire, establishing an axillary-femoral “through-and-through” guidewire on which the axillary sheath was advanced from above. Our approach is faster compared to that described by Antonello and Coll., since it avoids the first “snaring” inside the aneurysmal sac. Furthermore, in case of thrombus-filled aneurysm sacs, the 4F Ber catheters carry a lower risk of peripheral embolization compared to greater calipers of the catheters (i.e., that described by D’Elia et al. [6]) or to the need to perform a “double-snare,” as described by Antonello et al. [11].

The “through-and through” axillary-femoral guidewire offers the advantage of facilitating the antegrade advancement of the transaxillary sheath, straightening the curvatures at the descending aorta, giving stability to the sheath and reducing the manipulation of the arch, especially in the case of a “shaggy” aorta. This point has been described as a key strategy to reduce the risk for stroke in branched endovascular repairs [4]. In addition, a catheter can be advanced parallel to the “through-and-through” axillary-femoral guidewire without losing access, in order to cannulate the origin of the target vessel with greater stability.

With our technique, a transfemoral balloon dilatation of the branch can also be retrogradely performed in case of incomplete opening or external compression at the ostium.

Indeed, we have begun using this technique because of an incomplete right renal branch opening in a patient in our experience, for whom a surgical approach was then performed to complete the procedure. Thereafter, our technique has been used in all patients whenever feasible, i.e., with a good caliper of the femoral vessels.

A possible disadvantage of our method is the need for a double puncture of the main access and the need for at least a 9F sheath for the secondary access, to have enough space for both the 4F Pigtail and Ber catheters. This issue should be kept in mind when accessing vessels that are small or severely diseased.

However, in our case series, we did not record any access-related complication. Of course, our experience is limited by the small sample size, which prevents from generalization of the results.

On the other hand, when access vessels are permissive, the use of this technique has been proven to be a valid aid in our experience, and it can be considered at the beginning of the procedure, when the two Ber catheters can be advanced from both femoral arteries inside the aneurysmal sac and used in case of need for difficult cannulation of any branch.

## 5. Conclusions

In our experience, although limited, the placement of two catheters from both femoral arteries into the aneurysmal sac at the beginning of the procedure was a successful bailout technique for retrograde cannulation of any branch of the endograft and a “through-and-through” guidewire axillary-femoral technique when antegrade transaxillary cannulation was challenging or time-consuming.

## Figures and Tables

**Table 1:** Demographics and preoperative details with comorbidities of the patients of the case series.

Pt	Sex	Age	Pathology	Diameter (mm)	Urgent Repair	Active/Past Smoke	CAD	Hypertension	DM	Dyslipidemia	COPD	Renal Failure	Previous Abdominal Aortic Surgery
#1	m	78	TAAA type II	60	n	1	0	1	1	1	1	1	1
#2	f	82	TAAA type III	67	n	1	0	1	1	1	0	0	1
#3	m	80	pararenal AAA	59	y	1	1	0	0	0	1	0	0
#4	m	83	pararenal AAA	58	n	0	0	1	1	0	1	0	0

Pt = patient; TAAA = Thoraco-Abdominal Aortic Aneurysm; AAA = Abdominal Aortic Aneurysm; n = no; y = yes; CAD = Coronary Artery Disease; DM = Diabetes Mellitus; COPD = Chronic Obstructive Pulmonary Disease.

**Table 2:** Intraoperative details and in-hospital complications of the patients in the case series.

Pt	Type of Endografts	Branch Cannulated	Procedural Time (min)	Fluoroscopy Time (min)	Volume of Contrast Medium (mL)	Intraoperative Complications	Vascular Access Complications	In-Hospital Complications
#1	custom-made	LRA	245	112	198	0	0	permanent renal failure
#2	off-the-shelf	LRA	236	109	221	0	0	transient paraplegia
#3	off-the-shelf	CT	212	102	243	0	0	MI
#4	custom-made	RRA	197	102	201	0	0	none

LRA = Left Renal Artery; CT = Celiac Trunk; RRA = Right Renal Artery.

## Data Availability

Data will be available upon request to the corresponding author.
